# Endovascular Repair of a Dacron Pseudoaneurysm in an Ascending-to-Descending Aortic Bypass

**DOI:** 10.1055/s-0040-1715087

**Published:** 2020-12-11

**Authors:** Jacques Tomasi, Reda Belhaj Soulami, Marion Rolland, Jean-Philippe Verhoye

**Affiliations:** 1 Department of Thoracic and Cardiovascular Surgery, Pontchaillou University Hospital, Rennes, France

**Keywords:** aortic coarctation, aneurysm, endovascular repair, pseudoaneurysm

## Abstract

In the setting of postcoarctation aortic repair, Dacron graft dilatation and late aneurysms are not uncommon. Reintervention usually involves redo open surgery and replacement of the aneurysmal graft or the pseudoaneurysmal suture line. The present case describes the endovascular repair of a Dacron anastomotic false aneurysm in an extra-anatomic ascending-to-descending aortic bypass, 19 years after surgical correction of aortic recoarctation.

## Introduction

Late complications after aortic coarctation repair are not uncommon. 1 2 In fact, Dacron graft dilatation and late aneurysms have been reported as potential concerns at long-term follow-up. 1 2 3 4 5 In this setting, reintervention is reported in up to 10% of cases after coarctation repair, 2 usually involving redo open surgery and replacement of the aneurysmal graft or the pseudoaneurysmal suture line. 1 2 3 5 6 We herein describe a case of endovascular repair of a Dacron graft dilatation and subsequent anastomotic false aneurysm with an unusual anatomic aspect 19 years after surgical correction of aortic recoarctation by extra-anatomic ascending-to-descending aortic bypass.

## Case Presentation

A 39-year-old man with a history of aortic coarctation repair was transferred to our department for acute chest pain. At the age of 9 years, he underwent first coarctation repair through left posterolateral thoracotomy with aortic resection and graft interposition. He subsequently required a second procedure 11 years later due to recoarctation. An ascending-to-descending aortic extra-anatomic bypass was performed through a right posterolateral thoracotomy using two 20-mm Dacron grafts sewn in an end-to-end fashion.

**Fig. 1:**
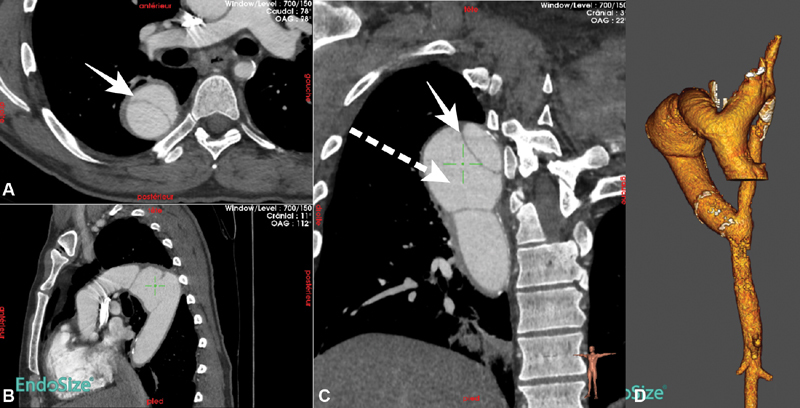
Preoperative computed tomography scan findings with three-dimensional (3D) reconstruction, revealing dilatation of the Dacron graft and a pseudoaneurysm of the end-to-end anastomosis between the two Dacron grafts. ( **A** ) Axial view, ( **B** ) coronal view, ( **C** ) frontal view, and ( **D** ) 3D reconstruction. Continuous arrow, prosthetic flap; dashed arrow, pseudoaneurysm.

Computed tomography revealed significant dilatation of the Dacron graft and a pseudoaneurysm of the end-to-end anastomosis between the two Dacron grafts with an unusual aspect of prosthetic flap ( Fig. 1 ). There was no clinical argument for septic false aneurysm. Immediate management of pain and blood pressure was started, with the target of lowering systolic blood pressure to 100 to 120 mm Hg. Preoperative planning demonstrated global dilatation of the Dacron grafts at 28 to 30 mm with an anastomotic false aneurysm measured at 57 mm ( Fig. 1 ). Redo surgery was deemed to carry high operative risk, and thus, endovascular treatment was chosen.

**Fig. 2:**
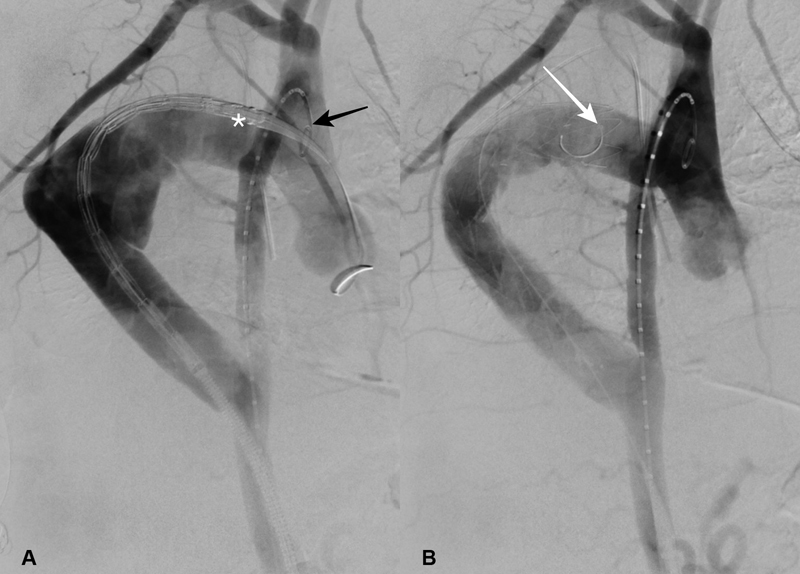
( **A** ) Perioperative fluoroscopic view. Initial angiography through the pigtail catheter in the ascending aorta confirmed the Dacron dilatation and false aneurysm. ( **B** ) Control angiography demonstrates successful implantation and false-aneurysm exclusion. Figure 8 markers: white (*) pigtail catheter in the ascending aorta; black arrow, pigtail catheter inside the ascending aorta; proximal figure 8 markers distal from the proximal anastomosis once the stent graft is delivered.

**Fig. 3:**
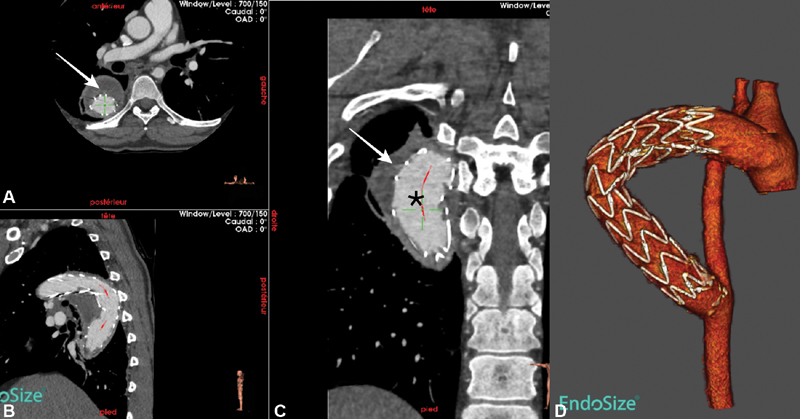
Postoperative computed tomography scan findings with three-dimensional (3D) reconstruction, confirming false aneurysm exclusion and thrombosis, correct positioning of the stent-graft, and the absence of endoleak. ( **A** ) Axial view, ( **B** ) coronal view, ( **C** ) frontal view, and ( **D** ) 3D reconstruction. White arrow, thrombosed pseudoaneurysm; black (*), patent stent graft.

Under general anesthesia, in a hybrid operating room, bilateral percutaneous femoral access was obtained after double Proglide (Abbott Scientific, Abbott Park, IL) arterial closure device insertion. A pigtail catheter was inserted through the left common femoral artery up to the ascending aorta for angiography ( Fig. 2A ). A 32-mm Medtronic (Medtronic Inc., Minneapolis, MN) VALIANT (VAMF3232) stent graft was introduced percutaneously through the right common femoral artery, on an Extrastiff Lunderquist wire (Cook, Bloomington, IN). After angiography, the stent graft was implanted under controlled hypotension. The Platinum iridium Figur8 markers were initially placed at the level of the proximal anastomosis ( Fig. 2A ). With the delivery system held stationary, the graft cover was withdrawn until two covered stents were exposed. The delivery system was subsequently withdrawn until the proximal end of the stent graft was more than 2 cm lower than the proximal anastomosis, ensuring that the proximal bare stents would not protrude inside the ascending aorta ( Fig. 2B ). Postimplantation angiography ( Fig. 2B ) demonstrated a satisfactory result, with successful deployment of the stent graft and false aneurysm exclusion. Control computed tomography confirmed the angiography results, with false aneurysm exclusion and thrombosis ( Fig. 3 ). The postoperative course was uneventful and the patient was discharged home 3 days after the procedure.

## Discussion

Dacron was first introduced and used as vascular substitute by DeBakey in 1953. In aortic surgery, modern-day Dacron grafts are made from knitted and woven fabric 2 and have high tensile strength and resistance to stretching and degradation. 3 Nonetheless, graft dilatation, true Dacron aneurysms, and anastomotic false aneurysms have been described at long-term follow-up. 3 5 6 In fact, Dacron grafts may dilate by up to 50% of their original size, 2 and most of the enlargement seems to occur within the first postoperative year. 2 In such cases, redo surgical repair carries significant mortality and morbidity, including paraplegia, bleeding, and paralysis of the recurrent nerve. 7

In the present case, progressive Dacron dilatation has led to a false aneurysm of the end-to-end anastomosis between the two Dacron grafts, 19 years after initial implantation. Despite some concerns regarding potential inadequate resistance of the degenerated Dacron against the radial force of the stent graft, the feasibility of endovascular repair in postcoarctation repair pseudoaneurysms has been suggested in other reports. 4 7 The choice of a Medtronic VALIANT endograft with proximal FreeFlo configuration allowed an accurate proximal implantation, avoiding stent-graft protrusion in the ascending aorta or jumping back in the Dacron bypass. The tip capture–release handle provides simple turn-and-pull motion to release proximal stents accurately. Minimal (10%) oversizing was applied to ensure an optimal seal while avoiding Dacron injury or pseudoaneurysm rupture during implantation. The device also demonstrates an enhanced conformability, flexibility, and kink resistance.

This report highlights the requirement for patients undergoing aortic coarctation repair to undergo long-term imaging follow-up of the aorta as well as the implanted Dacron grafts. At long-term, Dacron grafts may indeed develop true aneurysms, pseudoaneurysms due to the remaining diseased aorta, or, as illustrated in this case, pseudoaneurysms between two Dacron grafts, due to progressive graft failure. Finally, our report also demonstrates the feasibility of stent-graft implantation in degenerated Dacron grafts. Such approach may, however, not be suitable for all cases of Dacron failure, and long-term follow-up remains critical to ensure graft stability. To the best of our knowledge, this is the first report of endovascular treatment of an ascending-to-descending aortic bypass due to Dacron dilatation and subsequent anastomotic false aneurysm.
